# Investigating the potential protective effects of natural product quercetin against imidacloprid-induced biochemical toxicity and DNA damage in adults rats

**DOI:** 10.1016/j.toxrep.2019.07.007

**Published:** 2019-07-22

**Authors:** Abdel moniem S. Hassan, Fatma I. Abo El-Ela, Ayman Moustafa Abdel-Aziz

**Affiliations:** a Department of Biochemistry, Faculty of Agriculture, Fayoum University, Egypt; b Department of Pharmacology, Faculty of Veterinary Medicine, Beni-Suef University, Beni-Suef 62511, Egypt; c Department of Zoology, Faculty of Science, Fayoum University, Egypt

**Keywords:** Imidacloprid, Protective, Quercetin, Toxicity, Rats

## Abstract

Quercetin (QT) is a natural antioxidant materials that’s possesses different type of pharmacological activities. In the current study, the protective effect QT against imidacloprid (IMD)-induced toxicity in rats was studied. The experiment included thirty-six adult male rats groups treated with QT, IMD (two different doses), their combinations and control non-treated group for 21 consecutive days. Different biochemical analysis (serum liver and kidney enzymes level, cholesterol and Glucose levels) were evaluated. DNA damage using comet assay and histopathological examination of different body organs were also screened. Treatment with IMD increased ALT, AST, serum urea, creatinine, cholesterol and Glucose levels but decreased the levels of serum total protein, albumin and body weight with induction in triacylglycerol and cholesterol levels. Animals treated with QT prior to IMD administration showed normal enzymatic levels which indicating a protective effect of QT. In addition, QT protected the different body organs from the histological changes and DNA damages induced by IMD toxicity. The present results showed the protective effect of QT as a natural material against the IMD induced toxicity at different doses.

## Highlights

• Imidacloprid insecticide causes hepatotoxicity, renal damage and DNA damage.
• Quercetin revealed a significant protective action against the toxic effects of Imidacloprid.
• Quercetin counteracts the imidacloprid effects on liver, Kidney and DNA damage to the normal level.

## Graphical abstract

**Figure:**
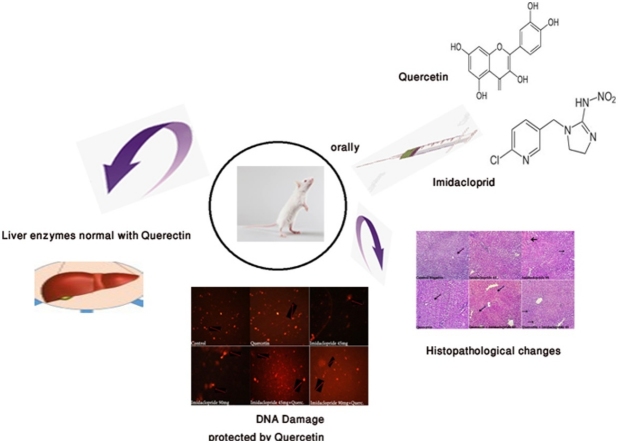

## 1 Introduction

Imidacloprid (IMD) is one of the main insecticides in our life that’s used for eradication of insects [1]; besides the other application of IMD in soil and seed dressing [2]. IMD acts as insecticide through inhibition the release or formation of acetylcholine through its Para sympatholytic effect. Different studies illustrate the IMD toxicity through variant mechanisms and effects on different organs as heart, kidney, nervous system and or even death. Recently; IMD toxicity effects on the immune system [3,4]. In addition to IMD reproductive toxicity in male rats [5] and [6].

In the previous studies; stated that hepatotoxicity is one of the main side effects for the IMD toxicity due to liver is the main organ for detoxification of pesticides and flavonoid metabolism [7].

IMD side effects not target only specific type of species but affect all different species as aquatic animals, animals and human. One of the main harmful effects of IMD is leading to carcinogenic and harmful mutagenic effects in both animals and human. Besides; oxidative stress and genotoxicity in specific species affects in addition to; the hepatotoxicity and nephrotoxicity at a dose much lower than the LD_50_ in mice. Previous multiple studies about IMD toxicity had been investigated [[8], [9], [10], [11], [12], [13], [14]]).

Quercetin (QT) is a flavonoid of plant origin which distributed in different vegetables and fruits. The main effects of QT acts through its antioxidant activity through ROS scavenging [15]. Other activities like anti-inflammatory and anti-fibrotic properties [16].

The main objective of the present study was to evaluate the role of quercetin on liver, kidney, spleen toxicity induced by imidacloprid for 21days. To achieve this aim, rats were given Imidacloprid and/or quercetin for 21 days by oral gavage. From this study quercetin as a natural antioxidant flavonoid has an important protective effect against imidacloprid toxicity.

## 2 Materials and methods

### 2.1 Experimental animals and treatment protocol

Total number of 36 adult male albino rats (average body weight 150 to 250gm) has been used in this study. Rats obtained from animal unit, faculty of veterinary medicine, Beni-Suef University. All normal conditions of acclimatization had provided for the animals as 50% humidity, 22 ± 2 °C temperature and 12 ± 1 h light-dark with feeding on standard diet and freely access of water According to the committee of animal's rights as this work was conducted with the formal approval of the local animal care committees and the clinical trials have been registered as legislation requires in Beni-Suef University, so animals treated ethically throughout the experiment study.

Rats were divided into six equal groups (6 rat/group) as follow:

Group 1 served as the control.

Group 2 treated with Quercetin (QT) at the dose of 100 mg/kg body weight.

Group 3 treated with both imidacloprid (IMD) 45 mg/kg and Quercetin (100 mg/kg).

Group 4 treated with both imidacloprid 90 mg/kg) and Quercetin (100 mg/kg).

Group 5 treated with imidacloprid at the dose of 45 mg/kg body weight].

Group 6 treated with imidacloprid at the dose of 90 mg/kg body weight.

### 2.2 Medications

Imidacloprid doses were selected based on its LD_50_, which is reported to be 450 mg/kg body weight [17]. Two doses, i.e., high dose, 1/5^th^ of LD_50_ (90 mg/kg) and low dose 1/10^th^ of LD_50_ (45 mg/kg) were selected. Imidacloprid 35% liquid form produced by *Cairo Company for chemicals*, Egypt. Quercetin powder was purchased from *Sigma Aldrich* as pure powder 98%. QT is a water-soluble compound; thus QT solutions were obtained by dissolving QT in sterile water. These drugs were administered by oral gavage every day consequently for 21 days. The imidacloprid was administered half an hour after administering of Quercetin. At the end of the experiment, rats were sacrificed by cervical dislocation after anesthesia with Ketamine (90 mg/kg.b.wt.) and Xylazine (5 mg/kg.b.wt.) combinations with ratio 1:1 and blood was collected without any anticoagulant. The serum separated was used for studying the serum biochemical analysis.

### 2.3 Estimation of the genotoxic effect on Liver DNA

Usually a portion of the left lateral lobe of the liver tissue is removed from a whole liver and then washed sufficiently with an ice-cold appropriate mincing buffer. The washed portion is minced to obtain the single cell suspension. The cell suspension is placed on ice to allow the cluster to settle down. Then, the supernatant can be used to make a comet slide. After preparing the comet slide, the slide is incubated with cold alkaline lysis solution overnight. After the lysis process, the slide is rinsed with deionized water to remove residual detergent and salts. After DNA unwinding using an electrophoresis solution, the slide is electrophoresed. After electrophoresis, the slide is neutralized. Then, the slide is dehydrated by absolute ethanol, air dried at room temperature.

#### 2.3.1 Evaluation of DNA damage

Ethidium bromide EtBr-stained DNA using 40 x objectives through the fluorescent microscope was used for Visualization of the DNA damage. Image analysis through the Komet 5 software was developed by kinetic imaging; Ltd. (Liverpool, UK) which was linked to CCD camera. This camera was used for estimation the qualitative and quantitative extent of DNA damage in the cells by measuring the length of DNA migration and the percentage of migrated DNA in 100 randomly selected cells per sample. Finally, the programme calculates the tail moment Fatma I. [18]

Tail moment = length of DNA migration (μm) X percentage (%) of migrated DNA.

### 2.4 Determination of biochemical parameters

Serum ALT, AST. Blood urea nitrogen (BUN), creatinine, Total protein, albumin, Total cholesterol and glucose were measured through Konelab20-fully automated biochemical analyzer (Thermo scientific, Japan) using standard diagnostic kits and analytical grade reagent [*Biosystems Egyptian Company for biotechnology (S.A.E), Egypt*] according to manufacturer’s instructions.

### 2.5 Histopathological examination

A total of 18 rats (3 rats/group) were randomly selected and euthanized and liver, Spleen, Kidney and Intestine were excised and fixed in 10% neutral buffered formalin. Samples were then routinely processed and embedded in paraffin wax. Sections (5 μm) were stained with hematoxylin and eosin (HE) [19,20]. The sections are then examined under light microscope at 200 and 400 × magnifications.

### 2.6 Statistical analysis

All data were statistically analyzed by one way analysis of variance (One-way ANOVA) and post comparison was carried out with LSD test using SPSS (Statistical Package for Social Sciences) version 17.00. The results were expressed as Mean ± SD and the values of *p < 0.05* were considered statistically significant [21]

## 3 Results

### 3.1 Effect of imidacloprid alone or pre-treated with quercetin on Body weight

**Fig. 1:**
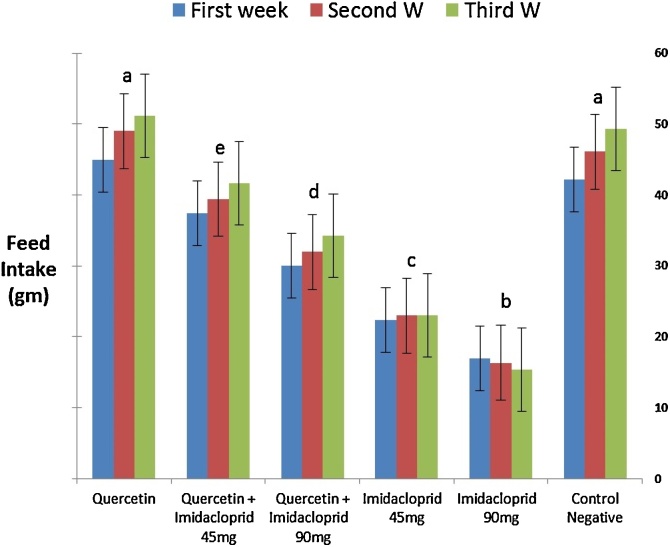
Effect of QT, IMD and their combinations on the Feed Intake levels. Each value represents the Mean±SEM of 5 animals with statistical significance lower than *p < 0.05*. Columns with the same letter showed no- significant difference. a: represents the significant difference at (*p* *<* *0.001*), b: represents the significant difference at (*p* *<* *0.01*) and c: represents the significant difference at (*p* *<* *0.05*).

**Fig. 2:**
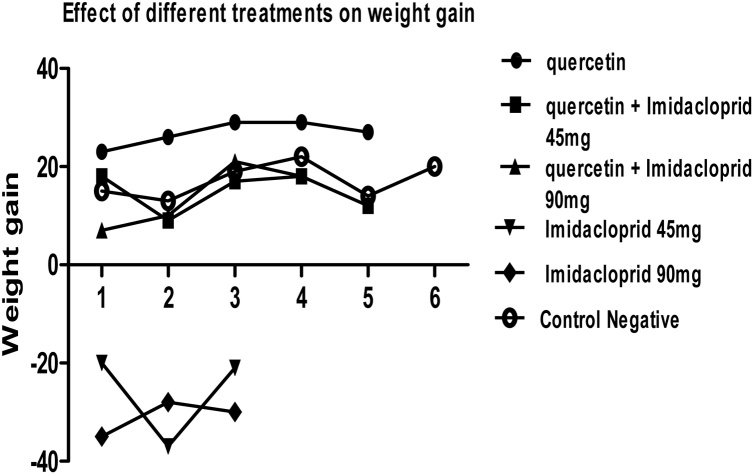
Effects of QT, IMD and their combinations at different doses on the weight gain by different treatment. Each value represents the Mean±SEM of 5 animals with statistical significance lower than *(p < 0.05)*.Imidacloprid (IMD) at different doses causes negative increase in the body weight with mortality so appeared here in the graph as negative results while the most increase in the body weight observed in the quercetin groups as appeared positive increase in the weight gain.

Data in (Fig. 1) presents the effect of different treatments on the adult rat's body weights. The maximum body weight observed in QT treated group when compared to other groups IMD or even pre-treated with QT. On the other hand gradual and significant (*p < 0.05*) decrease in the body weight in IMD treated groups (45 and 90 mg/kg b.wt) (with observed mortality in rats treated with 90 mg/kg b.wt.) when compared with control and other treated groups. Co-administration of quercetin combined with imidacloprid at different doses (group 3 and 4) showed a significant (*p < 0.05*) increase in the body weight as compared to the animals exposed to imidacloprid alone. However, no significant change was observed when the quercetin was given alone (group2) as compared to control. The weight gain of rats severely and significantly (*p < 0.05*) decreased in IMD treated groups at 45 and 90 mg/kg b.wt. (Fig. 2).

### 3.2 Serum hepatic enzyme activities

**Fig. 3:**
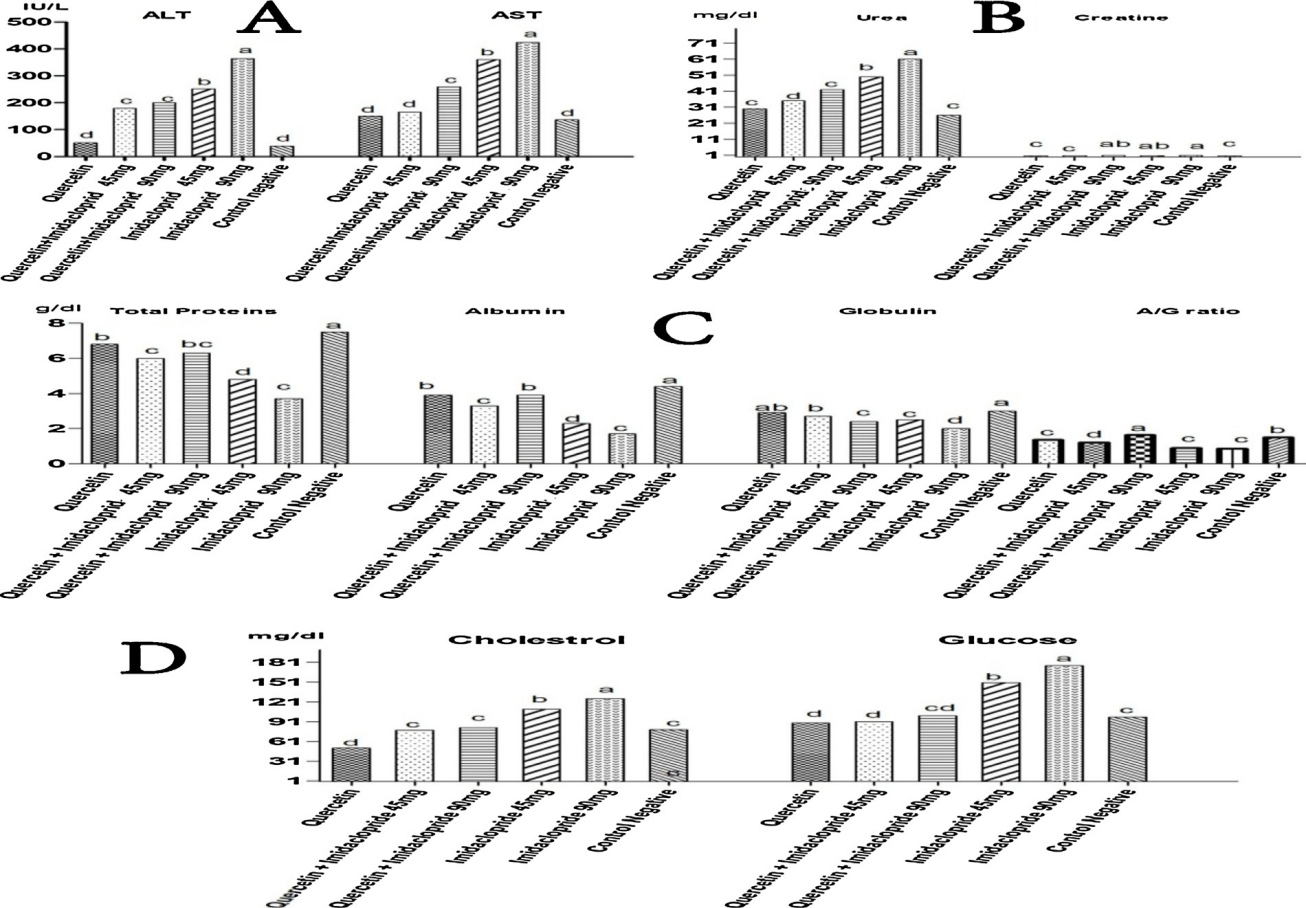
Effects of QT, IMD and their combinations at different doses on serum biochemical changes in adult rats. a: represents the significant difference at (*p < 0.001*), b: represents the significant difference at (*p < 0.01*) and c: represents the significant difference at (*p < 0.05*).

Data in (Table 1) shows that all liver biomarkers were significantly (*p < 0.05*) increased in dose dependent manner in the IMD-treated rats as compared to those of control rats after 21 days strongly suggesting the hepatotoxic effects of imidacloprid in rats. Co-administration of QT to the IMD -treated rats resulted in a significant (*p < 0.01*) partial recovery of the liver biomarkers, although AST and ALT activities were still significantly higher than those of the control rats at days 21. Besides QT treated group showed non-significant (*p > 0.05*) effects on liver enzymes when compared to control group (Table 1),(Fig. 3A).

**Table 1:** Effects of QT, IMD and their combinations on Serum biochemical activities in Adult rats.

A/G ratio	Globulin (g/dl)	Albumin (g/dl)	Total- protein (g/dl)	Glucose (mg/dl)	Cholestrol (mg/dl)	Creatinine (mg/dl)	Urea (mg/dl)	AST (IU/L)	ALT (IU/L)	Dose (mg/kg) b.wt	Group
1.50 ± 0.01^b^	3.0 ± 0.06^a^	4.4 ± 0.59^a^	7.5 ± 0.64^a^	98 ± 5.51^c^	79 ± 2.08^c^	0.57 ± 0.06^c^	26 ± 1.53^e^	137 ± 9.6^d^	39 ± 1.00^d^	0.1 ml/100g	Control Negative (CNT)
0.90 ± 0.03^e^	2.5 ± 0.10^c^	2.3 ± 0.15^d^	4.8 ± 0.25^d^	150 ± 2.08^b^	110 ± 8.50^b^	0.79 ± 0.09^ab^	50 ± 5.2^b^	360 ± 5.7^b^	252 ± 3.06^b^	45	Imidacloprid (IMD)
0.86 ± 0.03^e^	2.0 ± 0.10^d^	1.7 ± 0.15^e^	3.7 ± 0.25^e^	176 ± 4.4^a^	126 ± 5.51^a^	0.90 ± 0.01^a^	61 ± 1.53^a^	442 ± 6.6^a^	364 ± 1.00^a^	90	Imidacloprid (IMD)
1.36 ± 0.05^c^	2.9 ± 0.15^ab^	3.9 ± 0.10^b^	6.8 ± 0.23^b^	89 ± 1.00^d^	51 ± 3.21^d^	0.60 ± 0.10^c^	30 ± 1.00^e^	150 ± 1.53^d^	51 ± 1.53^d^	100	Quercetin (QT)
1.21 ± 0.03^d^	2.7 ± 0.15^b^	3.3 ± 0.10^c^	6.0 ± 0.25^c^	91 ± 7.21^d^	78 ± 3.51^c^	0.76 ± 0.05^c^	35 ± 1.00^d^	165 ± 1.6^d^	179 ± 4.73^c^	100 45	(QT) Quercetin (IMD) Imidacloprid
1.64 ± 0.06^a^	2.4 ± 0.06^c^	3.9 ± 0.10^b^	6.3 ± 0.12^bc^	100 ± 1.00^cd^	82 ± 2.00^c^	0.80 ± 0.10^ab^	42 ± 1.53^c^	259 ± 5.4^c^	200 ± 10.60^c^	100 90	Quercetin (QT) Imidacloprid (IMD)

Means within the same column having the same or no superscript denote non−significant variation (*P > 0.05*).

a: represents the significant difference at (*p < 0.001*).

b: represents the significant difference at (*p < 0.01*).

c: represents the significant difference at (*p < 0.05*).

### 3.3 Plasmatic renal factors estimation

Urea (BUN) and creatinine (CR) concentrations were measured in the serum to monitor the toxic effect of IMD and the protective effect of QT. No statistically significant *(p > 0.05*) changes were observed in the group treated with QT alone compared with the control group (*P > 0.05*). The BUN and CR concentrations were significantly increased by 134% (*P < 0.01*) and 57.89% (*P < 0.01*), respectively, in IMD -treated group compared with the control group. However, the BUN and CR levels were significantly decreased by 38% and 11.11% in the QT/IMD group, respectively, compared with the IMD -treated group, (*P < 0.05*) (Table 1),(Fig. 3B).

### 3.4 Serum total proteins, albumin and globulin levels

Total protein, albumin, globulin, and A/G ratio were assessed to diagnose protein metabolism. The changes in total and individual protein levels are presented in (Table 1). Serum total proteins and Albumin levels significantly decreased in IMD treated groups than CNT (control) group (*P < 0.001*), QT (Quercetin) (*P < 0.01*) and (IMD/QT) at different doses (*P < 0.01*).On the same hand globulin levels (*P < 0.05*) also decreased in IMD treated groups when compared to other treated groups (Fig. 3C). On simultaneous treatment of QT along with IMD the total protein, albumin, and globulin levels were brought back to normal.

### 3.5 Total serum cholesterol and glucose levels

Serum total cholesterol and glucose levels were significantly increased *(P < 0.001)* in dose dependent manner in IMD administered rats compared to that of control animals. In rats co-treated with QT along with IMD the serum cholesterol and glucose levels were significantly reduced (*p < 0.05*) compared to rats treated with imidacloprid alone which indicates the modulator effects of QT. These levels were non-significant in rats treated with QT alone when compared to the control (Table 1),(Fig. 3D).

### 3.6 Comet assay of liver DNA (Genotoxicity)

**Fig. 4:**
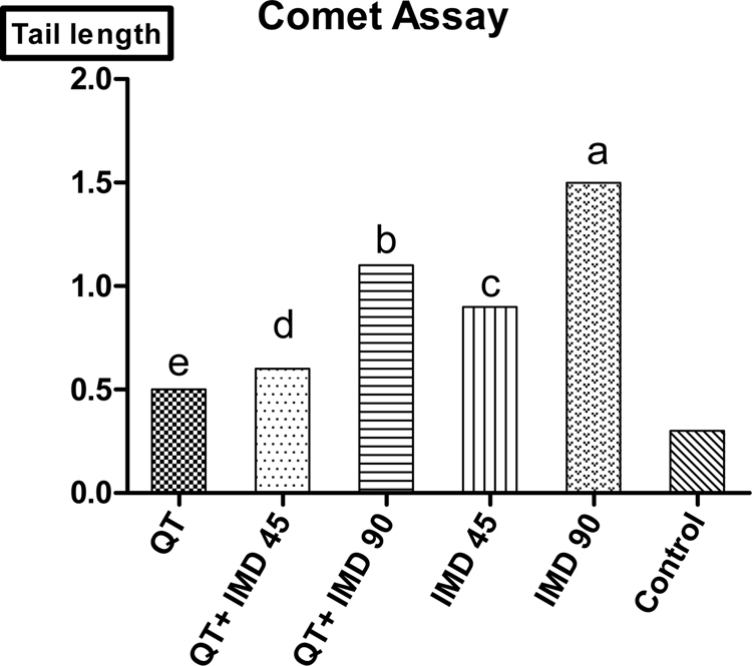
Effects of QT, IMD and their combinations at different doses on hepatic DNA (Genotoxicity) in adult rats (Mean; *N = 5*). The tail length indicative for the DNA damage which increased in IMD 90 mg and decreased when combined with quercetin and the same in MD 45 mg. a: represents the significant difference at (*p < 0.001*), b: represents the significant difference at (*p < 0.01*) and c: represents the significant difference at (*p < 0.05*).

**Fig. 5:**
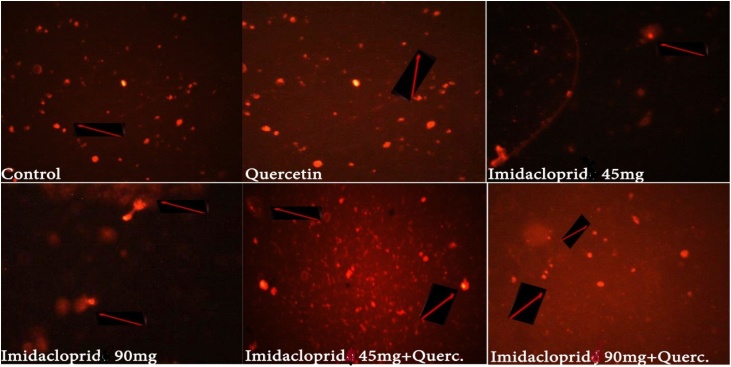
Effects of QT, IMD and their combinations at different doses on hepatic DNA (Genotoxicity) in adult rats. Both control and QT groups showed normal DNA of the hepatocytes without damage. In IMD treated groups, the damaged DNA is separated from the intact DNA (head) and generates a comet (tail) (red arrow) while QT combination with IMD showed intact cells with intact DNA.

A significant increase (*P < 0.01*) and (*P < 0.05*) in tail moment was recorded in IMD 90 mg when compared to the non-treated control and other groups respectively besides moderately reduced in the genotoxicity by its combination with QT, The tail length of the damaged DNA increased from 0.5 to 1.6 as showed on Fig. 4. On the other hand, QT group showed mild degree of hepatic genotoxicity and decreased the effect of IMD when compared to the non-treated control rats or IMD alone. IMD induced significant dose- dependent DNA SSB and oxidative DNA damage after exposure for 21 days. Quercetin treated group showed protective effects against damage in the liver DNA indicated by decreasing in the tail length when compared to IMD treated groups at different doses and also as shown in Fig. 4 which illustrates the increase in the tail length which indicative for the DNA damage in IMD and decreased by adding quercetin. Fig. 5 also clearly showed the appearance of the tail comet especially in IMD higher dose.

### 3.7 Histopathological findings

**Fig. 6:**
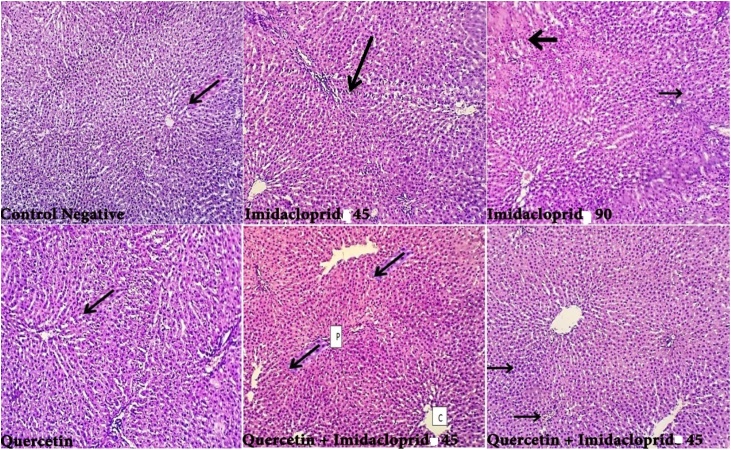
Liver histology of different treated groups in adult rats (H&E staining) showed normal histological structure and appearance in both Control and QT treated groups with normally appeared central vein (black arrow), normal hepatocytes and preserved lobular architecture. IMD treated groups showed hepatocytic hydropic degeneration and areas of centri-lobular necrosis (black arrow) which increase with dose. QT combinations with IMD markedly showed normal hepatocytes and preserved lobular architecture with unremarkable central veins (C) and portal tracts (p).

**Fig. 7:**
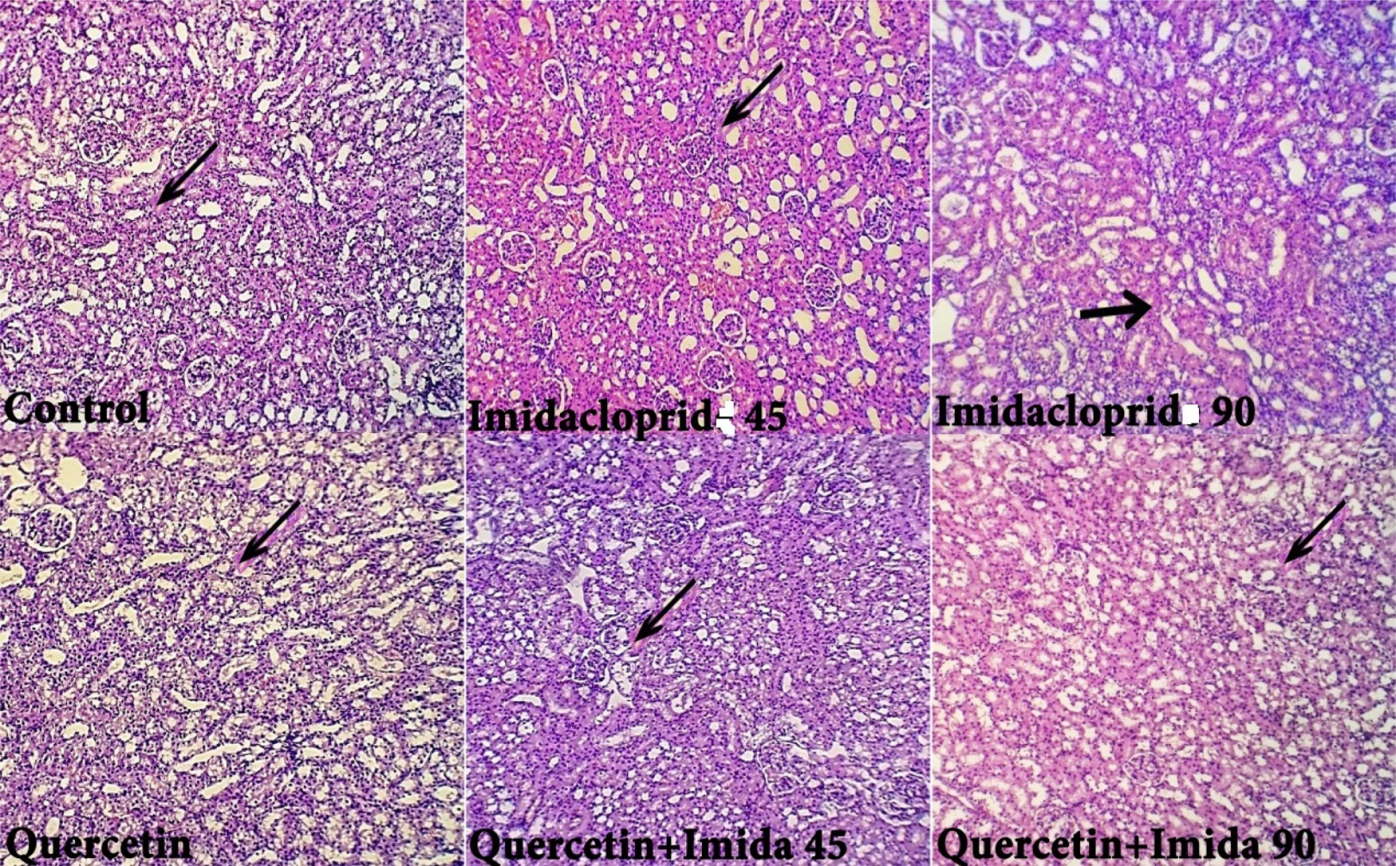
Histological examination of the spleen of both Control and QT groups showed normal white and red pulp with predominantly non-activated follicles and unremarkable red pulp in quercetin group only. IMD treated groups showed white pulp with moderate lymphocyte depletion (black arrow) which increase its severity with dose and moderately expanded red pulp. QT combination with IMD show normal white pulp and mildly expanded red pulp but moderately in QT/IMD 90 group.

**Fig. 8:**
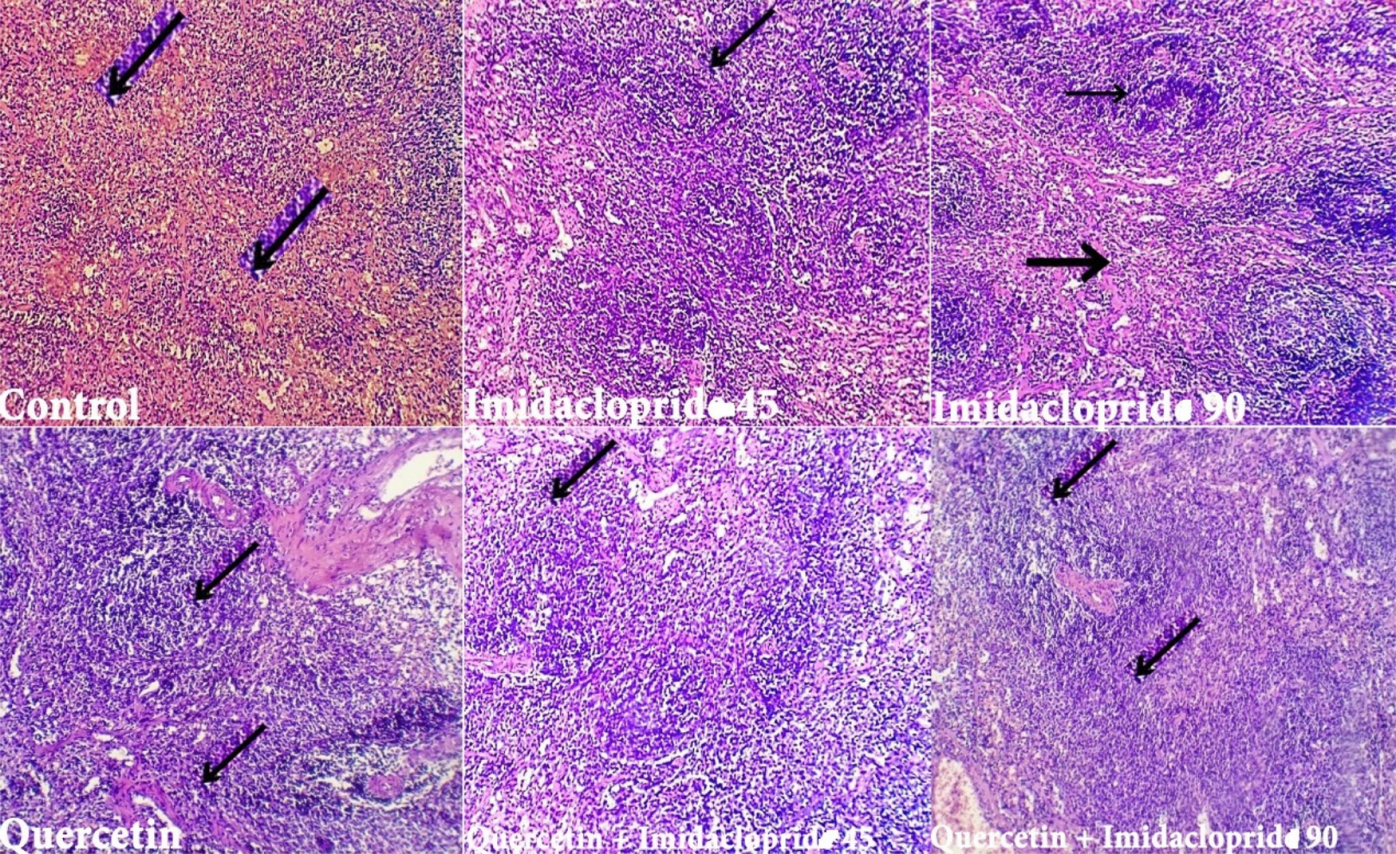
Histological examination of the Kidney showed regular glomeruli and renal tubules with moderate hydropic degenerative changes in QT group (black arrow). IMD treated groups showed renal tubules with focal necrosis increase its severity with dose increasing besides marked hydropic degenerative changes (black arrow). QT markedly protects the kidney from the destructive effect of IMD and showed renal tubules with mild hydropic degeneration and normal appeared structure.

**Fig. 9:**
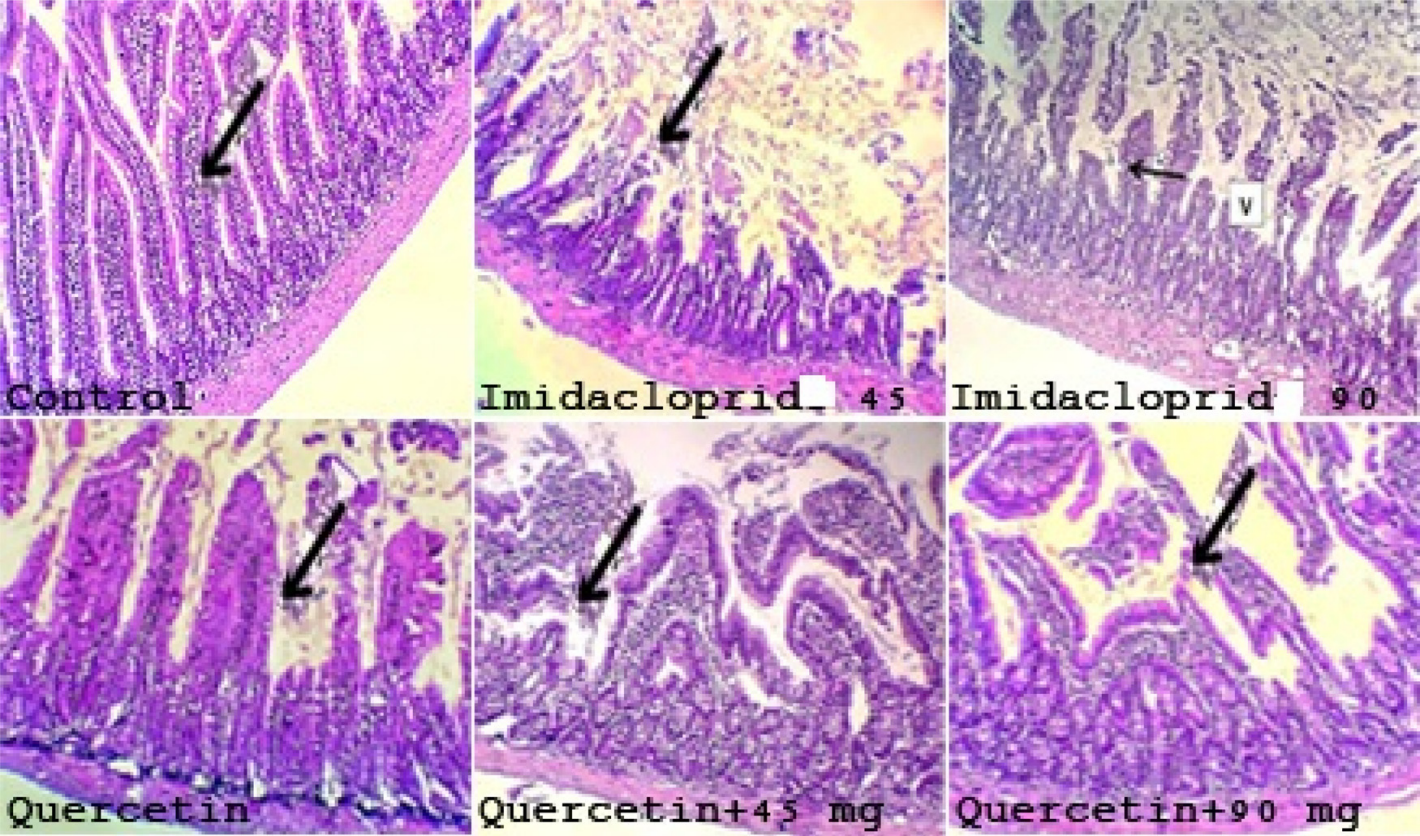
Histological examination of the intestine showed normal villous pattern, and minimal lamina propria inflammatory cells in both Control and QT (black arrow). Sections show mode rate villous broadening (V), and mild lamina propria inflammatory cells in IMD which increase with dose increase besides focal necrosis (black arrow). QT in combination with IMD showed mild villous broadening and mild lamina propria inflammatory cells.

To gain further insight into the tissue, we performed histology of the primary exposed area of the mice including liver and kidney. To check the toxicity caused by IMD we stained the tissue section by using H & E staining. Normal Liver histological structure was observed in both Control and QT treated groups with normally appeared central vein, normal hepatocytes and preserved lobular architecture. (Fig. 6) IMD treated groups showed hepatocytes hydropic degeneration and areas of centri-lobular necrosis which increases in a dose dependent manner. Quercetin combinations with IMD markedly showed normal hepatocytes and preserved lobular architecture with unremarkable central veins and portal tracts. Histological examination of the Kidney showed regular glomeruli and renal tubules with moderate hydropic degenerative changes in quercetin group (black arrow). IMD treated groups showed renal tubules with focal necrosis and its severity increases in dose dependent manner. In addition to marked hydropic degenerative changes (Fig. 7) black arrow). QT markedly protects the kidney from the destructive effect of IMD and showed renal tubules with mild hydropic degeneration and normal appeared structure. In the intestine and Spleen both showed normal structures in quercetin groups when compared to necrotic and inflammatory cells appeared in the imidacloprid group (Fig. 8, Fig. 9).

## 4 Discussion

IMD is an insecticide present in the tobacco and in different plants [22], acts on these insects through the Para sympatholytic effects [23]. IMD toxicity mainly through ingestion but of lower effect through inhalation or [24].

IMD has multiple applications for the control of sucking insects like ticks, white flies, rice hoppers, aphids, turf insects and termites. Besides also the IMD commonly used in cotton sugar beets, rice, soya beans, maize, potatoes, fruits and kitchen garden vegetables [25]

Recently multiple flavonoids plants have anti-oxidant activities through free radicals scavenging. One of the most abundant natural flavonoids, present in a large number of fruits and vegetables, is quercetin [26].

Quercetin is a natural material besides its antioxidant activities also chelates metal ions and act as lipid peroxidation inhibitor [27]. In addition; also quercetin showed other pharmacological activities as anti-diabetic, antitumor and anti-inflammatory [15]. Different investigations had been made in this study for exploring the possible protective effects of quercetin against the imidacloprid toxicity through measurements of different body functions, histopathological changes in rat liver, kidney, intestine and spleen in addition to genotoxicity to liver.

The results of the present study reveal that there was a reduction in body weight of rats treated with of imidacloprid. Despite unlimited access to food, there was a significant decrease in the body weight gain in male rats exposed to IMD for the period of 21 days. Significantly less weight gain and percent weight gain were noticed in IMD alone treated groups. The decrease in the body weight observed clearly in the IMD groups with dose increasing. This decrease negatively causes severe weight loss reached to about 80% in IMD 90 mg/kg b.wt., with mortality appear. On the other hand increase in the weight gain by about 70% in quercetin group and about 50% when combined with IMD. These findings are in accordance with previous workers [11,12]. Since weight gain in animals serves as an index of growth rate [28], decrease in weight gain observed in present study indicates toxic effect of IMD and might be attributed to the impairment in food assimilation due to tissue damage resulting in decreased absorption of nutrients from the gut.

Liver is the main active organ responsible for all xenobiotic detoxification [29]. Compromised liver and kidney functions by IMD administration have been clearly observed in present study. Imidacloprid at higher concentration caused significant increase in serum AST and ALT activities with rise in serum BUN and creatinine levels after 21 days exposure. Such elevation of serum AST and ALT activities as a result of IMD administration was documented by other authors [30]. Serum ALT and AST are considered to be among the most sensitive markers employed in the diagnosis of hepatotoxicity [31]. The increased AST and ALT were correlated with the histopathological changes in liver. During liver injury, transport function of the hepatocytes is disturbed which leads to leakage of plasma membrane, thereby causing an increased enzyme level in serum.

Moreover AST and ALT are indicative enzymes for liver damage in human [32] and animals [33]. Quercetin treated adult rats showed no adverse effects on liver enzymes that’s due to its antioxidant activity [34]. In addition Quercetin combination with imidacloprid (QT/IMD) also showed decrease in the elevating liver enzymes when compared with imidacloprid alone, Thus protective effects of quercetin due to its interaction with different radicals and scavenging them (peroxyl, superoxide, hydroxyl and alkoxyl) [35] also confirmed that Quercetin has a protective effect in adult rats. In the same ground Quercetin acts as antioxidant through its O-dihydroxy structure which gives it the higher stability against the free radicals so precipitating them in their delocalized electrons [36].

The increase in the creatinine and BUN levels in the IMD treated groups observed in this study may be due to the possible nephrotoxicity of IMD which indicate that IMD provoked nephrotoxicity. It can also be explained by histological observations that showed mild vacuolation in glomerular tufts and degeneration in the tubular lining epithelium in kidney. Significant increase of creatinine level in blood suggests evidence of marked impairment of kidney function [37].

IMD at different dose regimen causes also severe elevation in urea and creatinine serum enzyme levels which indicates renal damage and confirmed also by the histological examination, these results come in accordance with [38]. Oral administration of IMD for 21 days causes these changes in liver and kidney which come in accordance with [39] who confirmed that oral IMD for only 15 days sufficient for producing signiﬁcant toxic effects. On the same way Quercetin also protect the kidney from the damaged effects of imidacloprid due to its cytoprotectant effect in the kidney and prevention of the cellular injury and oxidative stress, and subsequently inhibited the leakage of renal enzymes into the blood circulation [40]. Histological analysis also confirmed the morphological changes in kidney exposed to IMD. High IMD dose resulted in more severe degenerative changes.

Imidacloprid treatment caused significant increase in serum cholesterol, glucose with decrease in total protein and albumin concentration in the present study. Similar findings were reported in other studies as a result of oral administration of different doses of imidacloprid [41]. This decrease in serum total protein may be due to lowered synthesis of albumin in liver in response to imidacloprid intake. It was reported that albumin levels are decreased in liver disease [42]. A decrease in globulin is expected as globulin (mostly γ-globulins) may be consumed in the production of antibodies in response to imidacloprid administration.

The increase of serum cholesterol level can be attributed to the effects of the pesticide on the permeability of liver cell membranes [43]. The rise in blood glucose might be an indication of disrupted carbohydrate metabolism due to enhanced breakdown of liver glycogen, possibly mediated by the reduced insulin activity. Pesticides may induce oxidative stress, leading to generation of free radicals and alteration in antioxidants, oxygen free radicals, the scavenging enzyme system, and lipid peroxidation and contributes to the toxicity [44]. Induction of excess production of ROS leading to alterations in the cellular antioxidant defense System and consequently effecting susceptibility to oxidative stress is one the main mechanisms of the action of many of the pesticides [45]. The free radical generation that leads to DNA damage, protein degradation, LPO and finally culminating into damage to various vital tissues like liver, kidney and brain [46]. These elevated free radicals and depressed antioxidant defense may lead to cell disruption, oxidative damage to cell membrane and hence increase susceptibility to LPO [47]. The damage of membrane lipids, protein and DNA are the endpoint biomarkers of oxidative stress-inducing toxic effect of pesticides [48]. Quercetin previously studied for its protective effects against DNA damage in human lymphocytes *in-vitro or in-vivo* ([[49], [50], [51]].

Quercetin prevents oxidant injury and cell death by several mechanisms, such as scavenging free radicals, donating hydrogen compound, quenching singlet oxygen and preventing lipid peroxidation [52] reported that quercetin is capable of protecting human leucocytes against oxidative DNA damage caused by hydrogen peroxide in a dose dependent manner. In the present study decreased level of ALT, AST, BUN and creatinine with restoration of histological architecture of liver and kidney were noticed in quercetin plus IMD treated animals as compared to imidacloprid alone treated rats. These findings are in accordance with the reports of some earlier workers who noted similar type of protection by quercetin against ethanol and poly-chlorine biphenyls induced toxicity in adult male Wistar rats [53]. In this study, it was found that quercetin attenuated the imidacloprid -induced DNA damage in liver cells.

On the other hand the protective effects of Quercetin abolish the oxidation of the hormone-sensitive lipase that’s responsible for lipid and cholesterol metabolism [54]. Oxidative damage to DNA can result in a number of different base oxidation states, which might have an impact on disease progression (cancer, cell death, carcinogenesis, and inflammation) [55]. In the previous studies, DNA damage was assessed using comet assay, which detected the breakup of DNA strand in each cell caused by free radicals [56]. In studying the genotoxic effects of imidacloprid by comet assay depends upon the fact that free radicals produced for pesticides and causes DNA damage [57,58]. On these area IMD treated groups Showed increase in the tail moment which indicator for the genotoxicity in the liver DNA. Pre-treatment with Quercetin as an antioxidant natural materials significantly protect the DNA damage due to its ability for decreasing the ROS generation thus prevents the pesticide induced derangement in the activities of the antioxidant enzymes [59].

Histological examination of the liver confirmed theses inflammatory changes and hepatotoxicity observed in IMD treated rats which come in accordance with [60] about imidacloprid hepatotoxicity. Quercetin-treated showed a normal liver architecture with decreasing the damaged effect of IMD when combined with it (Q. + IMIDA) these protective effects due to the antioxidant activity of quercetin for protection the hepatocyte from toxic free radicals [34].

## 5 Conclusions

This study has shown that Imidacloprid which is commonly used for the control of sucking insects causes hepatotoxicity and renal damage while co-administration of antioxidant quercetin reversed the hepatotoxicity, renal damage and prevents the DNA damage through its anti-oxidant activity. In the best of our knowledge; no previous study mentioned or studied the possibility of protect our crops, animals or even human form the IMD toxicity.

## Declaration of Competing Interest

All authors declare that there is no any conflict of interest.
